# Optimizing resource utilization for large scale problems through architecture aware scheduling

**DOI:** 10.1038/s41598-024-75711-8

**Published:** 2024-11-01

**Authors:** Ali M Elsawwaf, Gamal M Aly, Hossam M Faheem, Mahmoud Fayez

**Affiliations:** 1 Computer and Systems Engineering Dept, Faculty of Engineering, Ain Shams University, Cairo, Egypt; 2 Computer systems Department, Faculty of Computer and Information Sciences, Ain Shams University, Cairo, Egypt

**Keywords:** Parallel Processing, High performance computing, Task Scheduling, Engineering, Biomedical engineering, Electrical and electronic engineering, Mathematics and computing, Computational science, Computer science, Information technology, Computational biology and bioinformatics, Computational models, Computational platforms and environments, Data processing, Functional clustering, Genome informatics, Hardware and infrastructure, High-throughput screening, Programming language, Protein analysis, Sequence annotation

## Abstract

Rapid development realms of parallel architectures and its heterogeneity have inspired researchers to invent new scheduling strategies to efficiently distribute workloads among these architectures in a way that may lead to better performance. This paper presents a comprehensive study on optimizing resource utilization for large-scale problems by employing architecture-aware scheduling techniques. We conducted a series of experiments to measure the execution times of various architectures with different problem sizes. These experiments have been conducted multiple times to minimize measurement variance. The findings from these experiments are utilized to develop a scheduling strategy that enables faster completion of larger data-parallel problems while maximizing resource utilization. The proposed approach makes performance enhancement with 16.7% for large data size. It has a significant impact on enhancing computational efficiency and reducing costs in high-performance computing environments.

## Introduction

With the increasing complexity of computational problems and the exponential growth in number of independent tasks in high-performance computing, Optimizing capabilities and resource utilization has become a paramount concern. High-performance computing environments often involve the execution of large-scale problems that require significant computational resources. Allocating these resources efficiently while minimizing the time taken to complete tasks is essential for meeting computational demands, achieving cost-effectiveness, and maintaining competitiveness in various domains. The practical need to allocate resources based on previous system knowledge and ability to allocate different architectures to solve the computationally intensive problem requires a lot of trials to build such knowledge. This demand requires a well-established and defined resource attributes and algorithm library to choose the best implementations based on resources availability.

Challenges and Limitations: several challenges contribute to the complexity of the task scheduling problem for large-scale applications:

1. Heterogeneous architectures: Modern high-performance computing environments often consist of heterogeneous architectures with varying capabilities, including Central Processing Units (CPUs), Graphics Processing Units (GPUs), and specialized accelerators. Each architecture possesses a unique number of cores, processing power, memory bandwidth, and latency characteristics, making it challenging to efficiently allocate tasks across these diverse resources.
2. Varying problem sizes: Large-scale computational problems can span a wide range of sizes, from small-scale simulations to massive data-intensive tasks. A one-size-fits-all scheduling approach may lead to either underutilization or overloading of specific resources, resulting in suboptimal performance.
3. Measurement variance: Accurately measuring the execution times of computational tasks is essential for informed scheduling decisions. However, in real-world scenarios, execution times may exhibit variance due to system fluctuations, contention for resources, and other factors. Handling this variance and obtaining reliable measurements are critical for devising an effective scheduling algorithm.

Those challenges motivated us to build efficient scheduling strategy to distribute application workload chunks among different heterogeneous architectures such that the overall running time of the application is minimized. In this research, we address these challenges by conducting a set of carefully designed experiments to measure the execution times of various architectures for different problem sizes. By leveraging the experimental data, we propose an architecture-aware scheduling algorithm that allocates computational tasks to appropriate architectures, thereby optimizing resource utilization and minimizing job completion times.

The rest of this paper is organized as follows: "Problem statement" describes Problem Statement. "Related work of task scheduling strategies" describes the related work of task scheduling strategies and main contribution of the proposed algorithm. "Architecture-aware Scheduling Approach" represents the architecture-aware scheduling approach. "Experimental setup" presents the experimental setup, including description of the architectures used in the experiments, selection of problem sizes and benchmark application that ensure Real-world Relevance, Replicability and Comparability. "Experimental Results" presents Experimental Results that aim to get actual execution time of single task for different architectures. "Results Validation" validates the scheduling algorithm based on the experimental results. Finally, in "Conclusion" we conclude our work.

## Problem statement

Computationally intensive problems need enough computing power that can be provided by high performance computing (HPC) clusters. Currently, HPC clusters comprise of multi-core architectures, accelerators, coprocessors, and many other architectures. Each architecture has its own software framework. For example, NVidia GPGPUs use CUDA, Xeon-Phi coprocessors and traditional Intel CPUs can use MPI and OpenMP. Usually, HPC clusters’ users are not aware of the multi-core architectures and their programming paradigms. This always leads to over-subscribing of the HPC cluster resources. Accordingly, it leads to poor utilization of such valuable resources. This challenge requires a well-structured knowledge base of available H/W, S/W, and algorithms library in order to pick the most efficient way to carry on the users’ jobs on the cluster.

The heterogeneity of HPC clusters makes it very difficult to have a standard framework that can allow researchers and developers to implement their codes in a way to benefit from all the architectures existing in the cluster. This is because designing an execution model that can unify all computing resources is still very difficult^1–3^.

Working with heterogeneous architectures requires two things; the first is designing an execution model “runtime system” that unifies all computing units and associated embedded memory; while the second is developing and tuning powerful scheduling algorithms to allow scheduling of tasks to the available computing resources efficiently.

Scheduling Strategy is one of the major issues that affect the performance of a system comprising of heterogeneous architectures. The goal of the scheduler is to either scatter the jobs to make sure they finish in the shortest time possible or to stack them to maximize the number of concurrently running jobs.

In this paper, we focus on scheduling strategies for problems having a single instruction on multiple data items. Due to the nature of the operations required to implement such problems, an effective scheduling strategy can be suggested. The main idea benefits from the fact that the number of operations required to solve those problems can be divided into parts. Each part can work on a specific data size called “chunk”. Consequently, we can have a deterministic number of operations in almost all cases^1,4,5^.

## Related work of task scheduling strategies

Task scheduling problems for heterogeneous systems are more complex than that for homogeneous computing systems because of the different execution rates among processors and possibly different communication rates among different processors. Well-known scheduling strategies for heterogeneous architectures include Heterogeneous Earliest Finish Time (HEFT), Predict Earliest Finish Time (PEFT) and Critical Path of Processor (CPOP). The three strategies are examples of static scheduling strategies for heterogeneous architectures^6^. HEFT executes in 2 phases. In the first phase, the priorities of all tasks are determined. In the second phase, the task is allocated to the processor that provides the smallest value of earliest finish time (EFT) of the task. If communication cost weights of the task and computation costs of the same task on different processors are too large, HEFT is considered less reasonable for heterogeneous environment. PEFT maintains an optimistic cost table (OCT) which is denoted by a matrix used to rank tasks for processor selection. In OCT, rows represent tasks and columns represent processor. It chooses the minimum sum of computational cost and communication cost in all child nodes for task scheduling. PEFT loses its advantage in case of high parallelism and big communication data. In CPOP, all critical path tasks are assigned to the same processor, which leads to unbalance of processors and an increase in the schedule length^6,7^.

In addition, these algorithms still have the following drawbacks^7^:

- Most of them ignore the heterogeneity of different computing resources and different communication between computing resources.
- These algorithms make entry task duplication to all processors that lead to CPU overloading. Task duplication consumes more processing power and occupies processors that are used for other tasks.

**Fig. 1:**
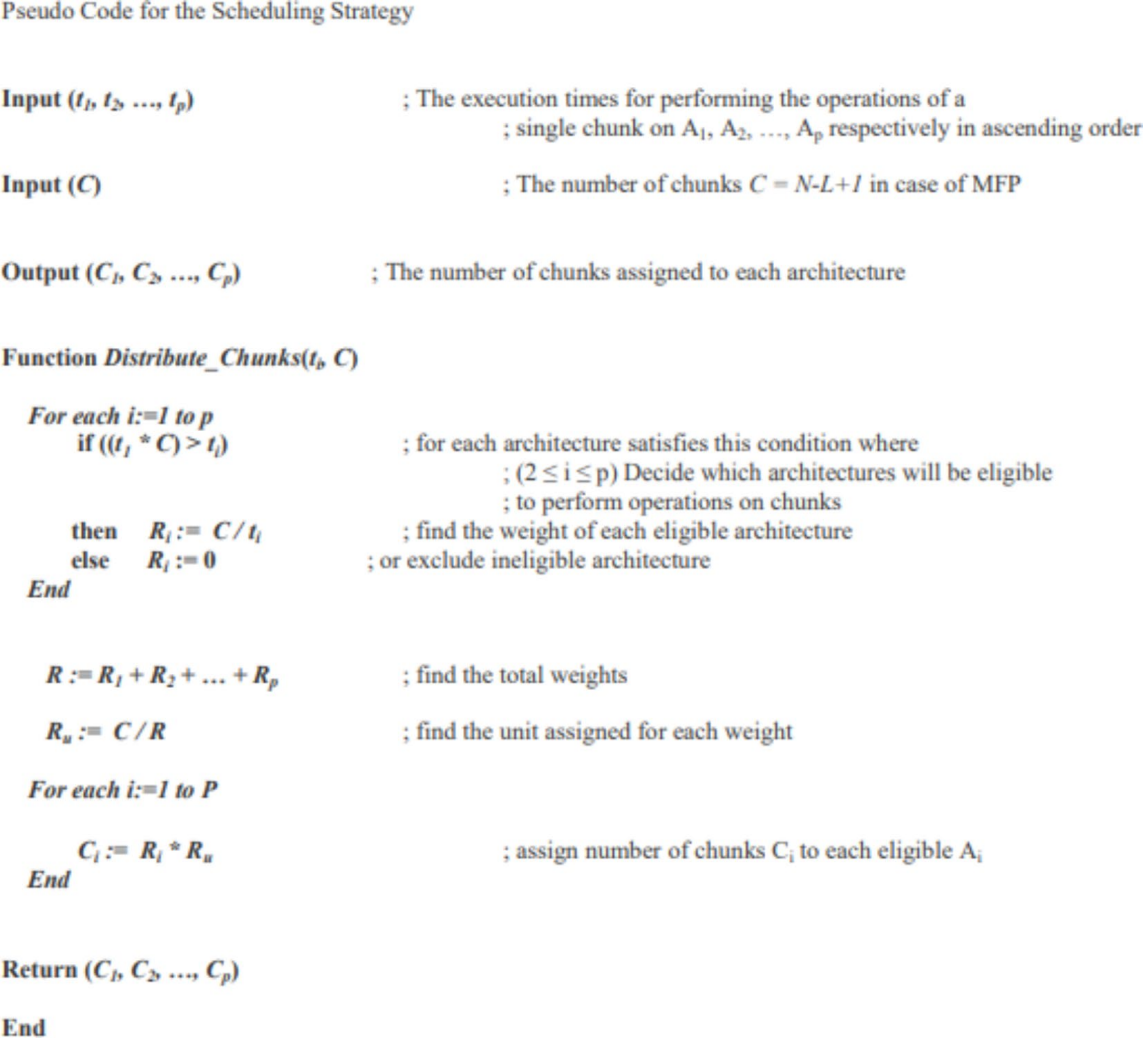
Pseudo code of scheduling algorithm ^8,9^ for solving Motif Finding Problem (MFP).

Speed-based scheduling strategy, which is displayed in Fig. 1, is recently used in scheduling^8,9^.

This strategy considers the speed of different architectures. It assumes that tasks of each chunk are executed by only a specific architecture. This assumption eliminates the factors of sharing resources that may affect the overall system performance. Faster architecture handles a larger number of chunks. Slower architecture gets smaller number of chunks that can be exactly processed in the same time granted to the fastest architecture. Initially, total execution time ($\:T$) required by the fastest architecture to handle all chunks, must be determined. Other slower architectures, that can’t handle at least one chunk in the same execution time ($\:T$), are excluded as ineligible architectures.

Speed-based scheduling strategy^8,9^ doesn’t consider that each time we add a new architecture to the fastest one; we get a new execution time $\:\left({T}^{{\prime\:}}\right)$, of the hybrid architecture, smaller than ($\:T$), which should be considered on the comparison process to determine and exclude other slower architectures. In addition, scheduling strategy^8,9^ doesn’t consider the actual execution time of a single task.

### Main contribution

In our proposed research, a modification to speed-based scheduling strategy is developed to solve computationally intensive problems on heterogeneous architectures efficiently. The proposed algorithm dynamically distribute workload among different architectures and discard ineligible architectures in more accurate way. The proposed algorithm considers both actual execution time of a single task and the new total execution time$\:\left({T}^{{\prime\:}}\right)$, of the hybrid architecture to exclude ineligible architectures. In "Architecture-aware Scheduling Approach" we propose steps and pseudocode of the proposed scheduling strategy. In Sections. "Experimental setup" and "Experimental Results" we apply required tests to deduce the optimum size of sample to get the actual execution time of a single task for each architecture. Results of these tests are used as inputs to the proposed algorithm. In "Results Validation" we validate the scheduling algorithm based on the experimental results in "Experimental Results". Results show that the proposed approach enhances performance with 16.7% for large data size.

This study focuses on splitting the job to the minimum number of resources to speed up the job and make sure it finishes with the smallest amount of time. In order to achieve this goal, the system must have a set of entities with well-defined attributes which are:

- Computing Resource (CR).
  - Number of Cores, RAM Size, CPU Speed, I/O Speed.
- Problem to Solve.
  - Problem Dimensions, Scalability Model in terms of the problem dimensions.
- Problem Solvers.
  - CR Compatibility, Configurations Parameters.

## Architecture-aware scheduling approach

**Fig. 2:**
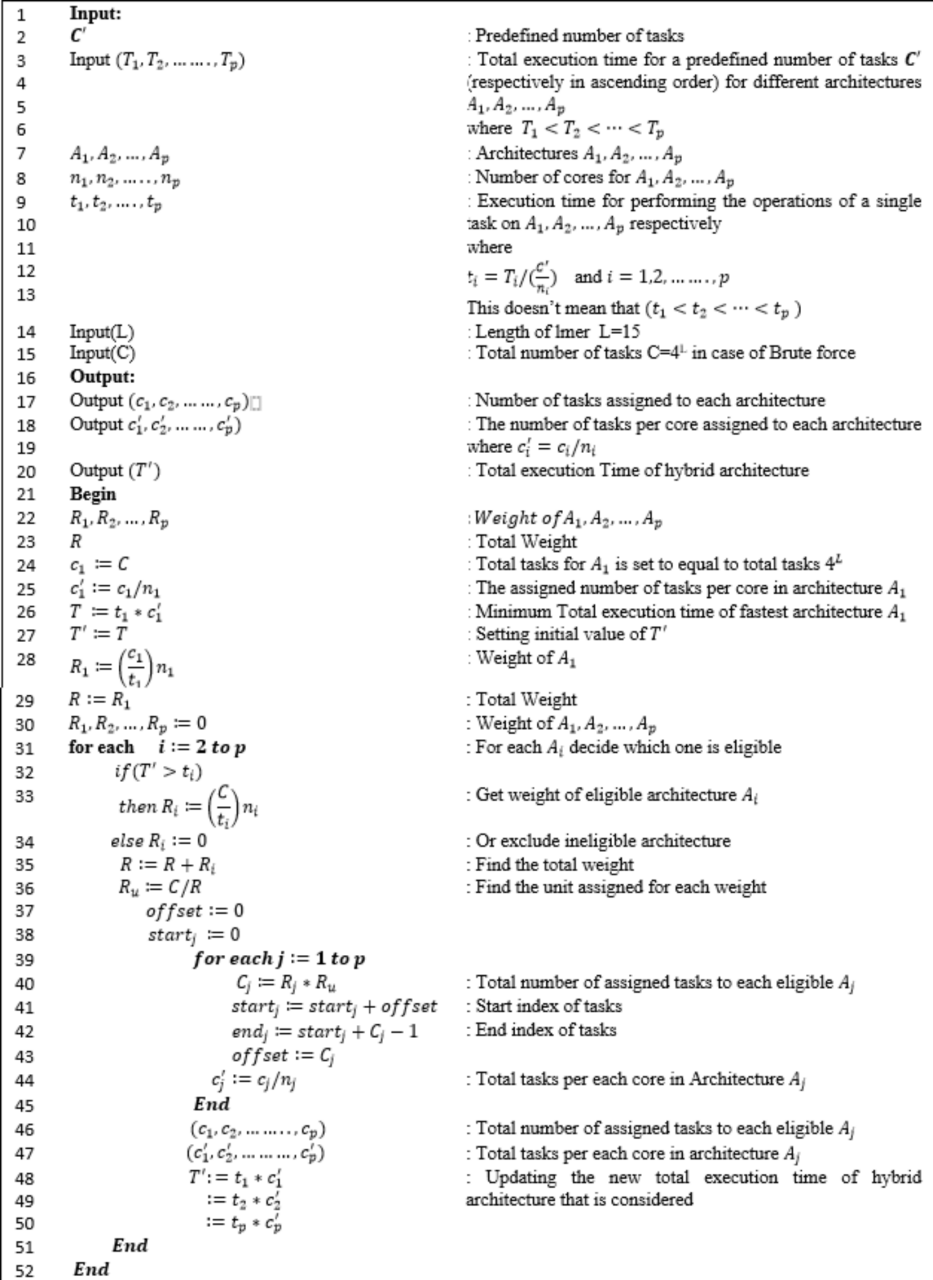
Pseudo code used in proposed Architecture-aware Scheduling Strategy.

**Fig. 3:**
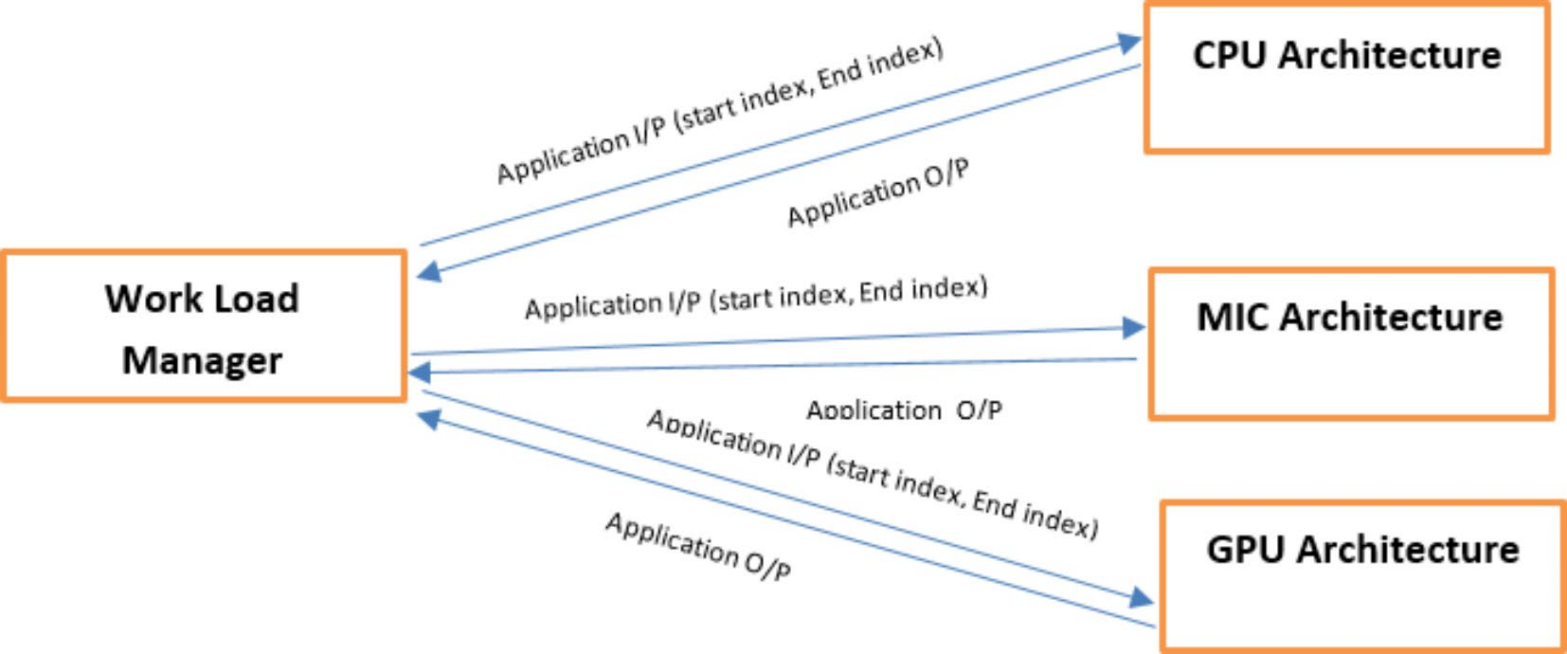
System Architecture of distributing workload.

This section describes the development of the scheduling strategy, declares its steps and pseudo code. In addition, it presents the development of the scheduling strategy that optimizes resource utilization for large-scale problems in high-performance computing environments. We provide a detailed explanation of the scheduling process and decision-making steps within the architecture-aware approach. Steps and pseudo code of proposed algorithm are displayed in Fig. 2. Figure 3 shows the system architectures and how the tasks are spawned on different architectures.

The strategy design is based on the following steps:

a. Considering total execution time for a given sample $\:{\varvec{C}}^{\varvec{{\prime\:}}}$ for CPU, GPU, and MIC architectures: Accordingly, architectures names $\:{A}_{1},{A}_{2},\dots\:,{A}_{p}\:$are sorted in ascending order according to total execution time $\:{T}_{1},{T}_{2},\dots\:\dots\:.,\:{T}_{p}$ of given sample $\:{\varvec{C}}^{\varvec{{\prime\:}}}$. Where $\:{T}_{1}<{T}_{2}<\dots\:<{T}_{p}$.

b. Considering actual execution time for a single task for different architectures: Considering parallel processing and number of cores in each architecture to be $\:{n}_{1},{n}_{2},\dots\:.,{n}_{p}\:$of $\:{A}_{1},{A}_{2},\dots\:,{A}_{p}$ respectively, the actual execution time $\:{t}_{1},\:{t}_{2},\dots\:\dots\:,\:{t}_{p}$ for performing the operations of a single task on $\:{A}_{1},{A}_{2},\dots\:,{A}_{p}\:$respectively

Where $\:{\varvec{t}}_{\varvec{i}}={\varvec{T}}_{\varvec{i}}/\left(\frac{{\varvec{C}}^{{\prime\:}}}{{\varvec{n}}_{\varvec{i}}}\right)$ and $\:\varvec{i}=1,2,\dots\:\dots\:.,\varvec{p}$ and $\:{\varvec{C}}^{{\prime\:}}$ = 40 M tasks.

Despite that the Total execution time array *T* is sorted ascendingly, this does not necessarily mean that $\:{t}_{1}<\:{t}_{2}<\dots\:<\:{t}_{p}\:\:$because the array *t* is normalized based on the number of cores per architecture.

c. For a given length of L-mer $\:\left(\:L\:\right)$, we get the total number of tasks $\:C=\:{4}^{L}$ in case of Brute force.

d. Considering new total execution of hybrid architecture: Starting from using the fastest architecture$\:{A}_{1}$, we investigate if the following architecture is eligible if its actual execution time for a single task ($\:{t}_{i}$) is less than the total execution time ($\:T={t}_{1}*\frac{C}{{\varvec{n}}_{1}}\:$) of $\:{A}_{1}$. In this case a new hybrid architecture results in new total execution time ($\:{T}^{{\prime\:}}$) where $\:({T}^{{\prime\:}}<T)\:$which ensures efficient use of available resources and minimizes job completion times. Instead of using $\:\left(T\right),$ new total execution time $\:{(T}^{{\prime\:}})\:$of hybrid architecture is used in comparison process to deduce if the next architecture is eligible.

e. Resource Constraints: The scheduling strategy considers various resource constraints, such as the number of available CPU, GPU, and MIC cores. The strategy optimally balances resource allocations to meet job requirements while maximizing system efficiency by minimizing the total execution time.

f. Scalability Considerations: The strategy considers the scalability of each architecture concerning problem size variations. It allocates larger tasks to eligible architectures with higher weight and smaller tasks to eligible architectures with smaller weight.

g. Real-world Case Studies: The effectiveness of the scheduling strategy is validated through real-world case study using practical problem sizes and MFP as a benchmark application. This case study demonstrates how the strategy optimizes resource utilization and reduces job completion times in realistic high-performance computing scenarios.

## Experimental setup

In the subsequent sections, we present the detailed description of the architectures used in the experiments, selection of problem sizes and benchmark application.

### Description of the architectures used in the experiments

In our research, we conducted experiments using a set of diverse computing architectures representative of the hardware commonly found in modern high-performance computing environments. Selected architectures were chosen based on their popularity, computational power, and widespread use in research and industry. Centos 7.9 is the running operating system, and Interconnection is IB (QDR) InfiniBand.

The selected architectures include:

#### Central Processing Units (CPUs)

CPU architecture consists of a total number of 20 nodes. Each CPU node has specifications as in Table 1.

#### Graphics processing units (GPUs)

We employ GPU node in Table 2 with many processing cores and high memory bandwidth to accelerate compute-intensive applications. These GPUs support CUDA and OpenCL programming models, allowing for efficient execution of parallel tasks.

#### Many integrated cores (MIC)

We deploy MIC node in Table 3. By integrating multiple cores onto a single chip, MIC processors offer massive parallelism and high computational efficiency, making them valuable tools for tackling complex computational challenges in various domains.

### Selection of problem sizes and benchmark application

To ensure the comprehensiveness and realism of our experiments, we carefully selected a range of problem sizes and benchmark applications that encompass a variety of computational complexities and reflect real-world scenarios.

#### Problem sizes

We chose problem sizes spanning a wide range, from small-scale to large-scale, to represent various computational workloads commonly encountered in high-performance computing environments. Small-scale problems provide insights into the performance of architectures when handling lightweight tasks, while large-scale problems challenge the capabilities of the computing resources for more intensive computations. The problem sizes were carefully chosen to be representative of typical data sizes and task complexities found in scientific simulations, data analytics, and machine learning applications.

#### Benchmark application

DNA Motif Finding Problem (MFP) was selected as a benchmark application as the number of independent tasks grows exponentially. The motif finding problem is a computational biology challenge focused on identifying short, recurring patterns called motifs within a set of related sequences, such as DNA, RNA, or proteins. These motifs are biologically significant and play essential roles in various cellular processes. The problem involves finding a motif of fixed length that appears in each sequence of the input set with few or no mismatches, subject to additional constraints like the maximum allowed number of errors. In this study we use classical MFP as a bioinformatics problem that represents different computational domains and that leverage the strengths of the architectures under study. By using MFP, we aimed at evaluating the performance of each architecture. In motif finding problem, given a set of sequences, each of the same length, the goal is to find a motif of fixed length (usually relatively short) that occurs in each sequence of the set with few or no mismatches^9–14^.

Motif finding algorithms can be categorized into two major groups, exact and approximate solutions^10,11,13^.

- Exact solution^15–18^:
- Apply exhaustive enumerations.
- Guarantee global optimality.
- Examples: Brute force, Skip Brute force, Recursive Brute force (R-BF).
- Approximate solution:
- Based on probability.
- Apply some form of local search.
- Doesn’t guarantee global optimal solution.
- Examples: Gibbs sampling and Expected maximization (EM).

**Table 1:** Regular CPU-based Compute Node.

Attribute	Value
Architecture	x86_64
CPU(s)	24
Thread(s) per core	1
Core(s) per socket	12
Socket(s)	2
CPU MHz	2399.852
Memory	96 GB

**Table 2:** NVIDIA CUDA compute Node.

Attribute	Value
(13) Multiprocessors, (192) CUDA Cores/MP	2496 CUDA Cores
GPU Clock rate	706 MHz (0.71 GHz)
Total amount of global memory	5120 MBytes (5368512512 bytes)
Total amount of constant memory	65,536 bytes
Total amount of shared memory per block	49,152 bytes
L2 Cache Size	1,310,720 bytes

**Table 3:** Xeon Phi Compute Node.

Attribute	Value
Total No of Active Cores	60
Voltage	897,000 uV
Frequency	1,052,631 kHz

#### Key components of the motif finding problem

a. Input: The input to the motif finding problem consists of a set of sequences ($\:S$), usually represented as strings of (N) characters over an alphabet (A, C, G, T for DNA sequences or A, C, G, U for RNA sequences, etc.), permitted mutation (d) and the desired motif length (L). The motif length indicates the number of characters (nucleotides or amino acids) that the motif should consist of.

b. Output: The output of the motif finding problem is the discovered motif, which is a string of characters of the specified length (L) that appears as a subsequence in every sequence of the input set ($\:S$), allowing for a certain number of (d) mismatches or errors (permitted mutation).

Accordingly, MFP has 5 dimensions D_1_, D_2_, D_3_, D_4_ and D_5_:

• $\:{D}_{1}$ is a set of sequences (Input sequence number) ($\:S$): This parameter represents the total number of sequences in the input dataset. As the number of sequences increases, the search space for potential motifs also grows, making the problem more complex and computationally demanding.

• $\:{D}_{2}$ is the length of each string (Input sequence length) (N): The sequence length refers to the number of characters (e.g., nucleotides or amino acids) in each individual sequence. Longer sequences can increase the problem size, as the potential number of motif occurrences grows with the sequence length.

• $\:{D}_{3}$ is a Motif length (L): The motif length is the number of characters in the motif pattern that algorithm aims to discover. Larger motif length generally increases the computational challenge, as longer motifs are less likely to occur frequently in the sequences.

• $\:{D}_{4}$ is permitted mutation (d): Larger permitted mutation generally increases the computational challenge. This parameter has no effect in case of using Brute Force, where all $\:\left({4}^{{D}_{3}}\right)$ Lmers are extracted.

• $\:{D}_{5}$ is a set of DNA alphabet (A, C, G, T)

c. Scoring: In some variants of the problem, scoring functions are used to quantify the quality of a candidate motif. The objective is to find the motif that maximizes the scoring function.

d. Computational Complexity: The motif finding problem is NP-hard, which means that finding an exact solution for large input sets is computationally infeasible in polynomial time^19–25^.

In this research, Brute force is applied, as an exact solution, to solve the Motif finding problem with the following dimensions: $\:{D}_{1}=20\:$strings, $\:{D}_{2}=600\:$characters/string, $\:{D}_{3}=15$ characters/motif, $\:{D}_{4}=4$ permitted mutations and $\:{D}_{5}=4$ in case of DNA alphabet (A, C, G, T) set. Brute force extracts all $\:\left({D}_{5}^{{D}_{3}}\right)$ that results in (4^15^) possible L-mers to be compared with all extracted motifs of each string sequence to find the motif that optimizes the scoring function.

Accordingly, MFP solver has a total number of comparison operations as in Eq. (1):

1 $$Total{\text{ }}Number{\text{ }}of{\text{ }}comparison{\text{ }}operations{\text{ }}=~\,\left( {D_{5}^{{{D_3}}}} \right)\,\, * {D_1}\,\, * \,\left( {{D_2} - {D_3}\,+\,1} \right)$$

$$= \left( {4^{{15}} } \right)\, * \,20\, * \,\left( {600 - 15 + 1} \right)$$

Where $\:{D}_{3}=15$ characters per each L-mer, $\:{D}_{2}=600\:$ characters per each string, and $\:{D}_{1}=20\:$ strings

#### Real-world relevance

The selected benchmark application has real-world relevance and is widely used in research and industry. It is used to evaluate the performance of high-performance computing systems. Using this widely recognized benchmark application allows us to draw meaningful comparisons with other studies and assess the practical implications of our architecture-aware scheduling approach.

#### Replicability and comparability

The choice of problem sizes and benchmark application ensures that our experiments are replicable by other researchers, enabling direct comparisons and validation of our findings. Replicability is crucial for establishing the reliability and generalizability of our results.

In the following section, we present measurement results for each selected problem size to deduce the minimum sample size of tasks and actual execution time of a single task for each architecture. The collected data serves as the foundation for our architecture-aware scheduling approach, enabling us to optimize resource utilization and minimize job completion times for large-scale problems in high-performance computing environments. The comprehensive evaluation of the proposed approach against the selected benchmark provides valuable insights into its effectiveness and practicality for real-world applications.

## Experimental results

In this section, we present the experimental results and regression models found, which include the execution times for different architectures and problem sizes. In this research we conduct a set of trial runs with different problem size dimensions to build a regression model for each problem solver. In order to find out the accurate model for each problem we must conduct the trails till the variance of the measured time tends to zero. To get the actual execution time of a single task we choose problem size to start from 1 M tasks to 10 M tasks with a step of 1 M and another problem size starting from 10 M tasks to 90 M tasks with a step of 10 M.

We start by investigating standalone CPU architectures to calculate the actual average execution time of a single task on single and multiple CPU nodes. In addition, actual average execution time of single task on both MIC and GPU architectures are investigated. These results are used in "Experimental Results" as inputs to the proposed scheduling algorithm.

**Fig. 4:**
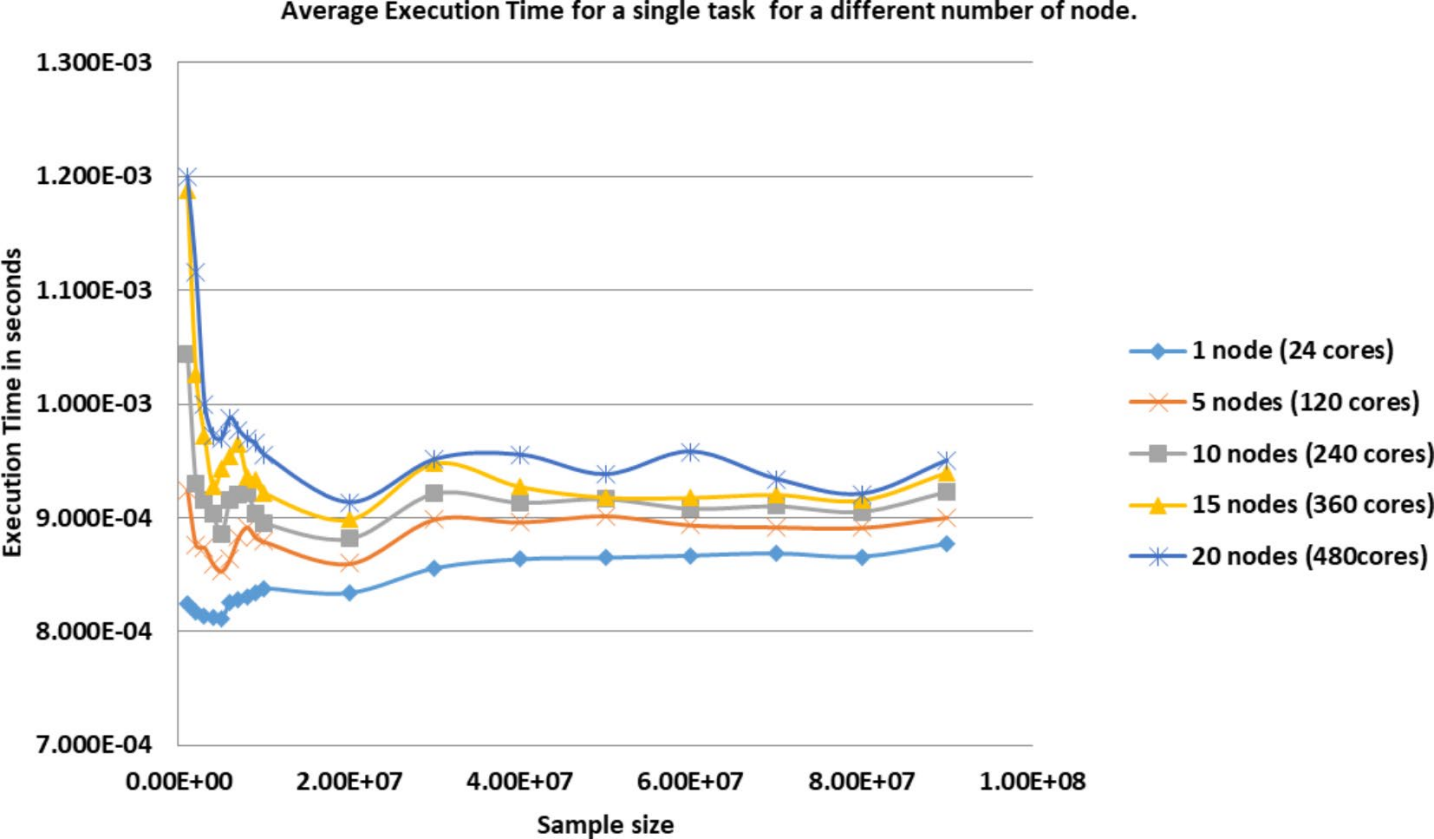
Average execution time for a single task (per core) in CPU architecture that consists of 1, 5, 10, 15 and 20 nodes.

Table 4 represents the average execution time of a single task for standalone CPU architecture that consists of 1, 5, 10, 15 and 20 nodes. Each sample in Table 4 represents the average execution time of 20 trials for a single task where variance tends to zero. This data is displayed in Fig. 4. Tables 5,6 represent average execution time and variance respectively with the best problem solver for DNA MFP using CPU, GPU, and MIC architectures respectively. MFP solver is a computational problem written in any programming language that supports the target architecture. Each solver must accept input parameters corresponding to each dimension of the problem along with two extra parameters to specify start and end indices of the problem size to allow scheduler to assign portion of the problem for each architecture.

**Fig. 5:**
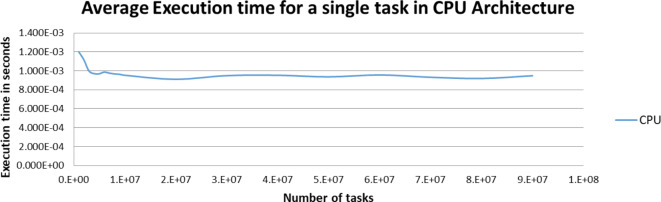
Average execution time for a single task per core in CPU architecture.

**Fig. 6:**
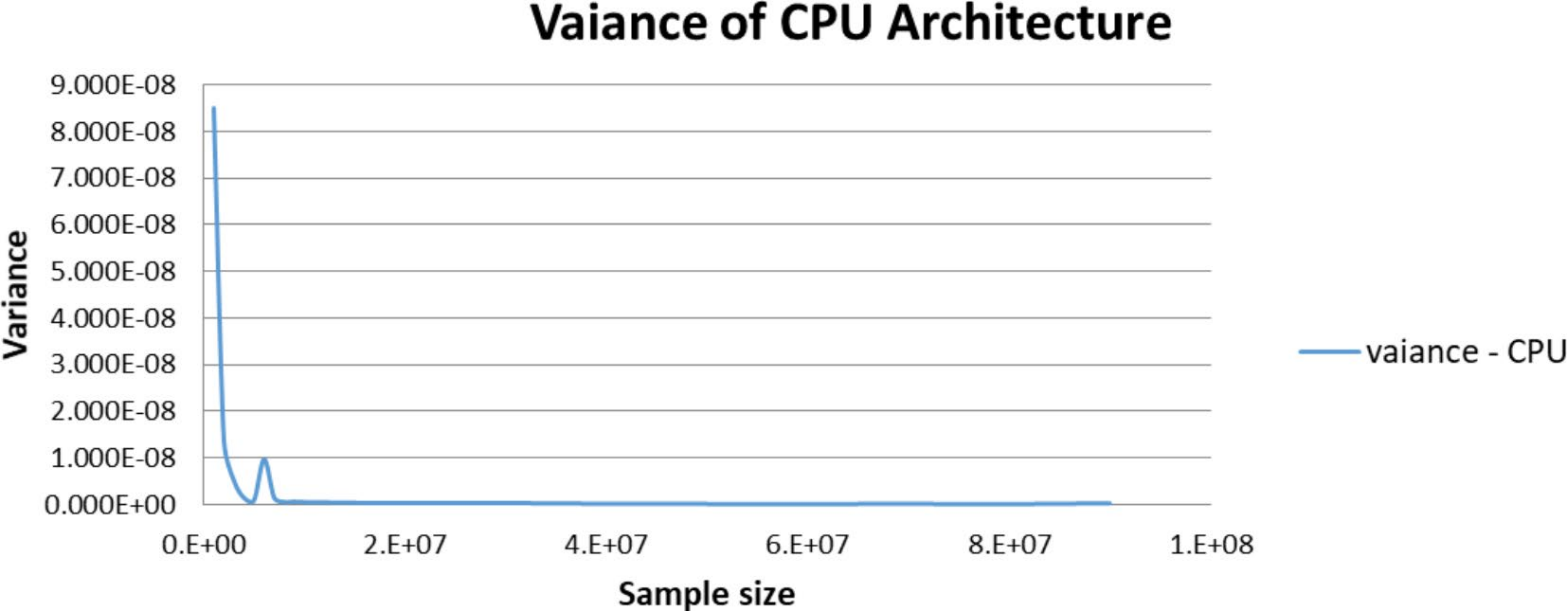
Variance of CPU architecture.

**Fig. 7:**
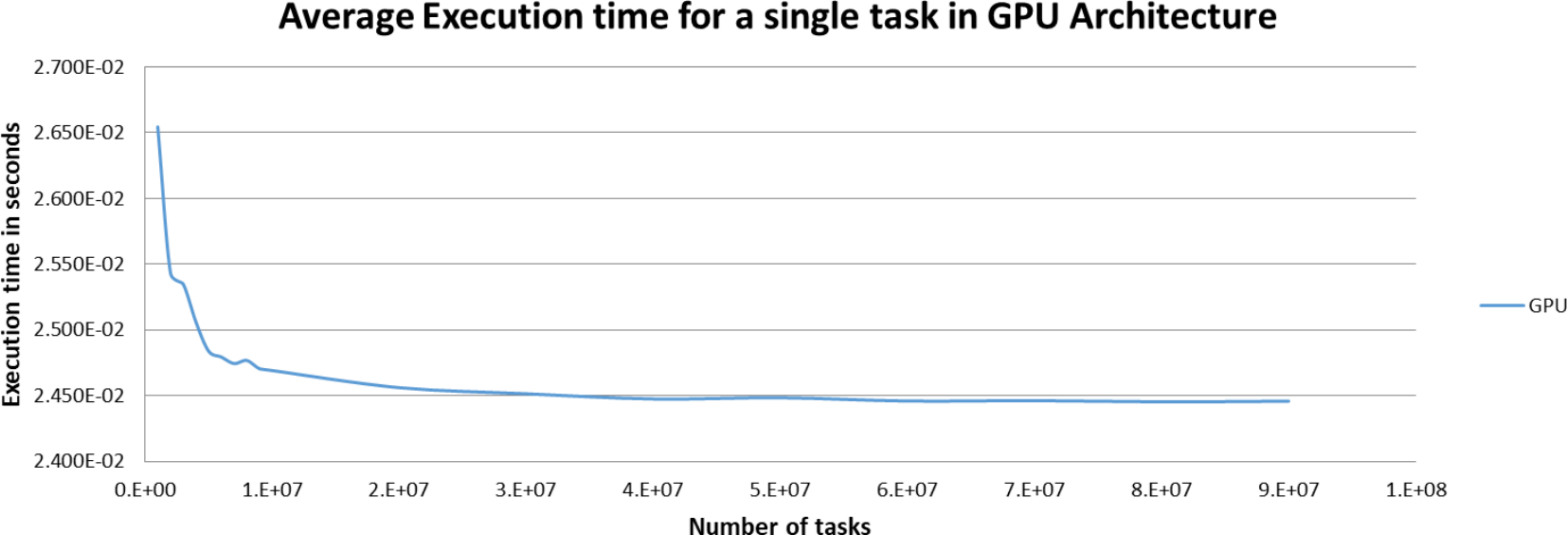
Average execution time for a single task per core in GPU architecture.

**Fig. 8:**
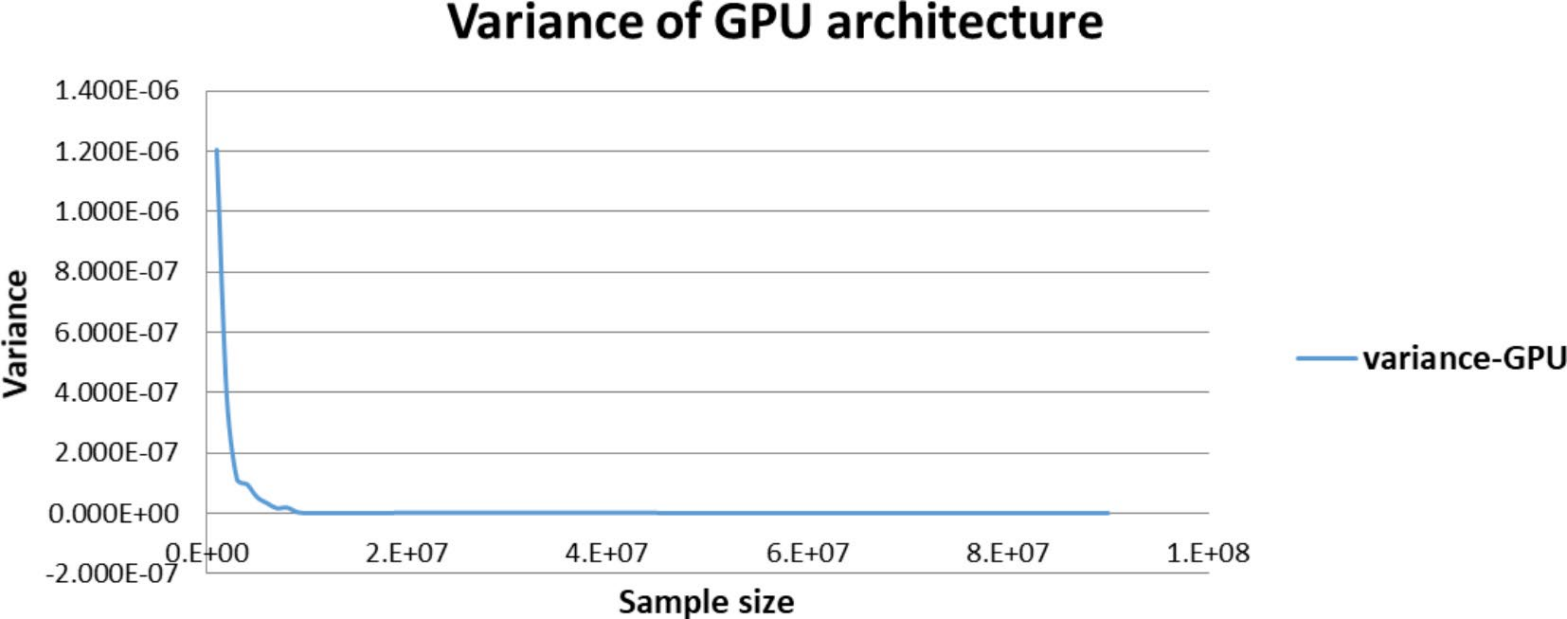
Variance of GPU architecture.

**Fig. 9:**
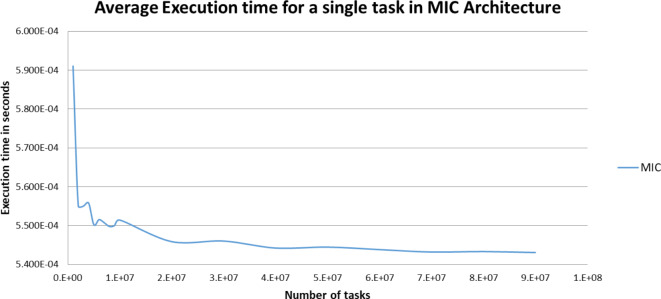
Average execution time for a single task per core in MIC architecture.

**Fig. 10:**
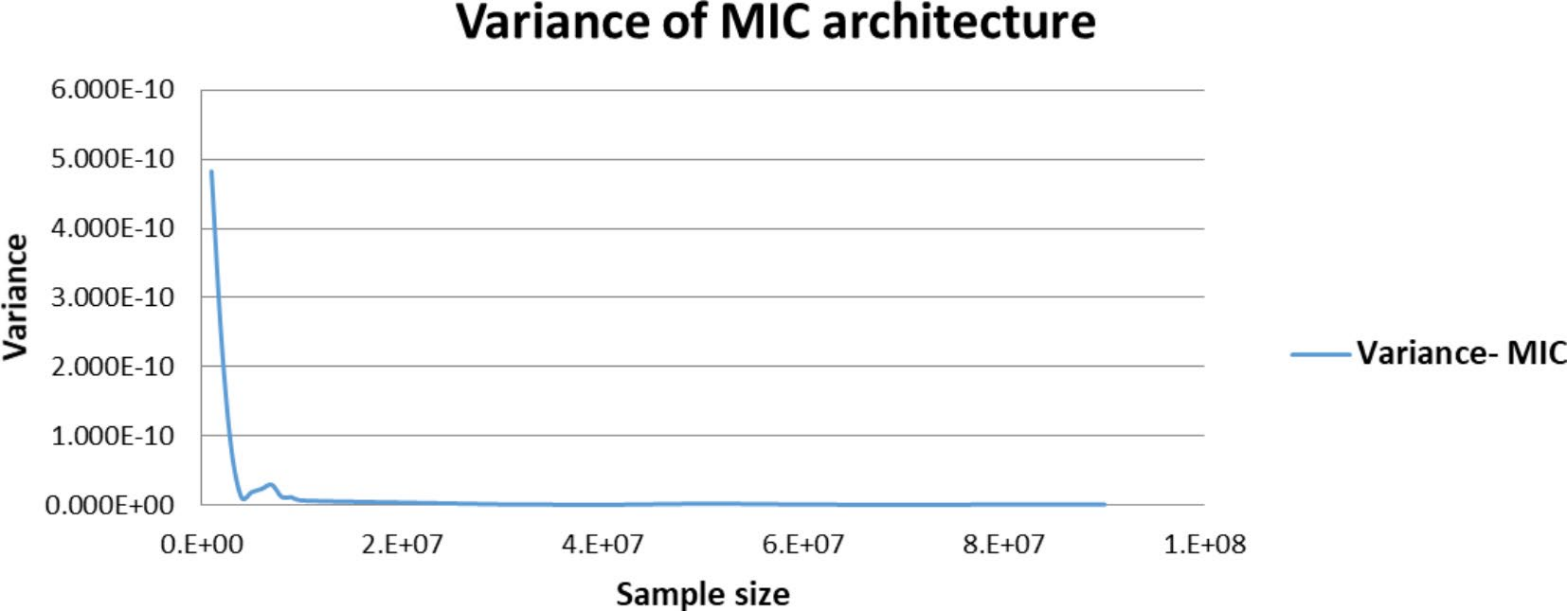
Variance of MIC architecture.

Figures 5,6,7,8,9,10 display average execution time for a single task per core and variance using CPU, GPU, and MIC architectures respectively. Based on Fig. 4, for the same problem size, execution time of a single task increases as the number of nodes (cores) increase due to communication overhead between master core on master node and distributed cores on different nodes that belong to the same CPU cluster. Accordingly, instead of using execution time of a single task in a single node, we consider actual execution time of a single task of CPU architecture that consists of 20 nodes as an input to the architecture-aware scheduling algorithm. Based on Tables 5,6 and Figs 5,6,7,8,9,10, problem size of 40 M tasks is chosen to be the optimum number for such problem to get the actual execution time of a single task where variance tends to zero and execution time of single task tends to be in steady state.

**Table 4:** Average execution time for a single task per core for CPU architecture.

#Tasks	Average execution time (Seconds) for a single task				
	1 node (24 cores)	5 nodes (120 cores)	10 nodes (240 cores)	15 nodes (360 cores)	20 nodes (480cores)
1E + 06	8.244E-04	9.240E-04	1.044E-03	1.188E-03	1.200E-03
2.E + 06	8.166E-04	8.760E-04	9.300E-04	1.026E-03	1.116E-03
3.E + 06	8.132E-04	8.740E-04	9.160E-04	9.720E-04	1.000E-03
4.E + 06	8.127E-04	8.595E-04	9.030E-04	9.270E-04	9.720E-04
5.E + 06	8.107E-04	8.532E-04	8.856E-04	9.432E-04	9.696E-04
6.E + 06	8.258E-04	8.640E-04	9.160E-04	9.540E-04	9.880E-04
7.E + 06	8.280E-04	8.829E-04	9.206E-04	9.643E-04	9.771E-04
8.E + 06	8.298E-04	8.918E-04	9.210E-04	9.360E-04	9.690E-04
9.E + 06	8.343E-04	8.827E-04	9.040E-04	9.340E-04	9.653E-04
1.E + 07	8.378E-04	8.790E-04	8.952E-04	9.216E-04	9.552E-04
2.E + 07	8.338E-04	8.598E-04	8.814E-04	8.982E-04	9.132E-04
3.E + 07	8.556E-04	8.986E-04	9.212E-04	9.474E-04	9.512E-04
4.E + 07	8.635E-04	8.961E-04	9.132E-04	9.270E-04	9.552E-04
5.E + 07	8.647E-04	9.014E-04	9.163E-04	9.176E-04	9.379E-04
6.E + 07	8.666E-04	8.935E-04	9.078E-04	9.174E-04	9.580E-04
7.E + 07	8.686E-04	8.918E-04	9.101E-04	9.201E-04	9.339E-04
8.E + 07	8.657E-04	8.912E-04	9.053E-04	9.151E-04	9.207E-04
9.E + 07	8.771E-04	9.002E-04	9.227E-04	9.394E-04	9.501E-04

**Table 5:** Average execution time in seconds for a single task per core for each architecture.

#Tasks	Average execution time (Seconds) for a single task		
	CPU */ xeon 480 cores	GPU* /K20 2496 Cuda cores	MIC* / xeon phi 60 cores
1.E + 06	1.2000E-03	2.6542E-02	5.9100E-04
2.E + 06	1.1160E-03	2.5431E-02	5.5500E-04
3.E + 06	1.0000E-03	2.5348E-02	5.5500E-04
4.E + 06	9.7200E-04	2.5060E-02	5.5575E-04
5.E + 06	9.7000E-04	2.4838E-02	5.5020E-04
6.E + 06	9.8800E-04	2.4793E-02	5.5150E-04
7.E + 06	9.7700E-04	2.4743E-02	5.5071E-04
8.E + 06	9.6900E-04	2.4767E-02	5.4975E-04
9.E + 06	9.6500E-04	2.4704E-02	5.5000E-04
1.E + 07	9.5500E-04	2.4690E-02	5.5140E-04
2.E + 07	9.1300E-04	2.4560E-02	5.4585E-04
3.E + 07	9.5100E-04	2.4513E-02	5.4600E-04
4.E + 07	9.5500E-04	2.4474E-02	5.4420E-04
5.E + 07	9.3800E-04	2.4483E-02	5.4444E-04
6.E + 07	9.5800E-04	2.4458E-02	5.4380E-04
7.E + 07	9.3400E-04	2.4461E-02	5.4317E-04
8.E + 07	9.2100E-04	2.4452E-02	5.4330E-04
9.E + 07	9.5000E-04	2.4457E-02	5.4303E-04

*Each sample in the table represents the average execution time of 20 trials for a single task where variance tends to zero.

**Table 6:** Variance for CPU, GPU, and MIC architectures.

#Tasks	Variance for CPU, GPU, and MIC		
	CPU / xeon 480 cores	GPU /K20 2496 Cuda cores	MIC / xeon phi 60 cores
1.E + 06	8.4884E-08	1.2032E-06	4.8316E-10
2.E + 06	1.3794E-08	3.8501E-07	2.3684E-10
3.E + 06	5.0526E-09	1.1408E-07	7.8947E-11
4.E + 06	1.3642E-09	9.6252E-08	1.1250E-11
5.E + 06	8.7309E-10	5.3901E-08	1.9326E-11
6.E + 06	9.6168E-09	3.3421E-08	2.3947E-11
7.E + 06	1.4230E-09	1.6697E-08	2.9968E-11
8.E + 06	4.8316E-10	1.8799E-08	1.2434E-11
9.E + 06	5.6889E-10	3.7629E-09	1.1696E-11
1.E + 07	4.6080E-10	5.0682E-35	7.2000E-12
2.E + 07	2.1069E-10	7.6200E-10	4.2395E-12
3.E + 07	2.5533E-10	9.0905E-10	1.6842E-12
4.E + 07	9.7011E-11	1.0026E-09	8.5263E-13
5.E + 07	4.9475E-11	4.1068E-10	2.7436E-12
6.E + 07	4.6316E-11	4.0551E-10	1.4316E-12
7.E + 07	7.7212E-11	3.2739E-10	9.3577E-13
8.E + 07	2.0747E-11	2.1055E-10	1.3263E-12
9.E + 07	2.2538E-10	1.9805E-10	1.3322E-12

**Table 7:** Average total (40 M) and single task execution time (sec) for different architectures.

#Tasks	Architecture		
	CPU / xeon (480) cores	MIC / xeon phi (60) cores	GPU /K20 (2496) Cuda cores
4.E + 07	79.6	362.8	396.5
1	9.550E-04	5.442E-04	2.447E-02

Results of single task execution times for different architectures and problem sizes serve as key inputs to the architecture-aware scheduling algorithm, which is designed to optimize resource utilization and minimize job completion times for large-scale problems in high-performance computing environments.

The minimized measurement variance provides a strong foundation for drawing meaningful conclusions and making informed decisions in the subsequent steps of the research, including the development of the architecture-aware scheduling approach and performance evaluation.

**Fig. 11:**
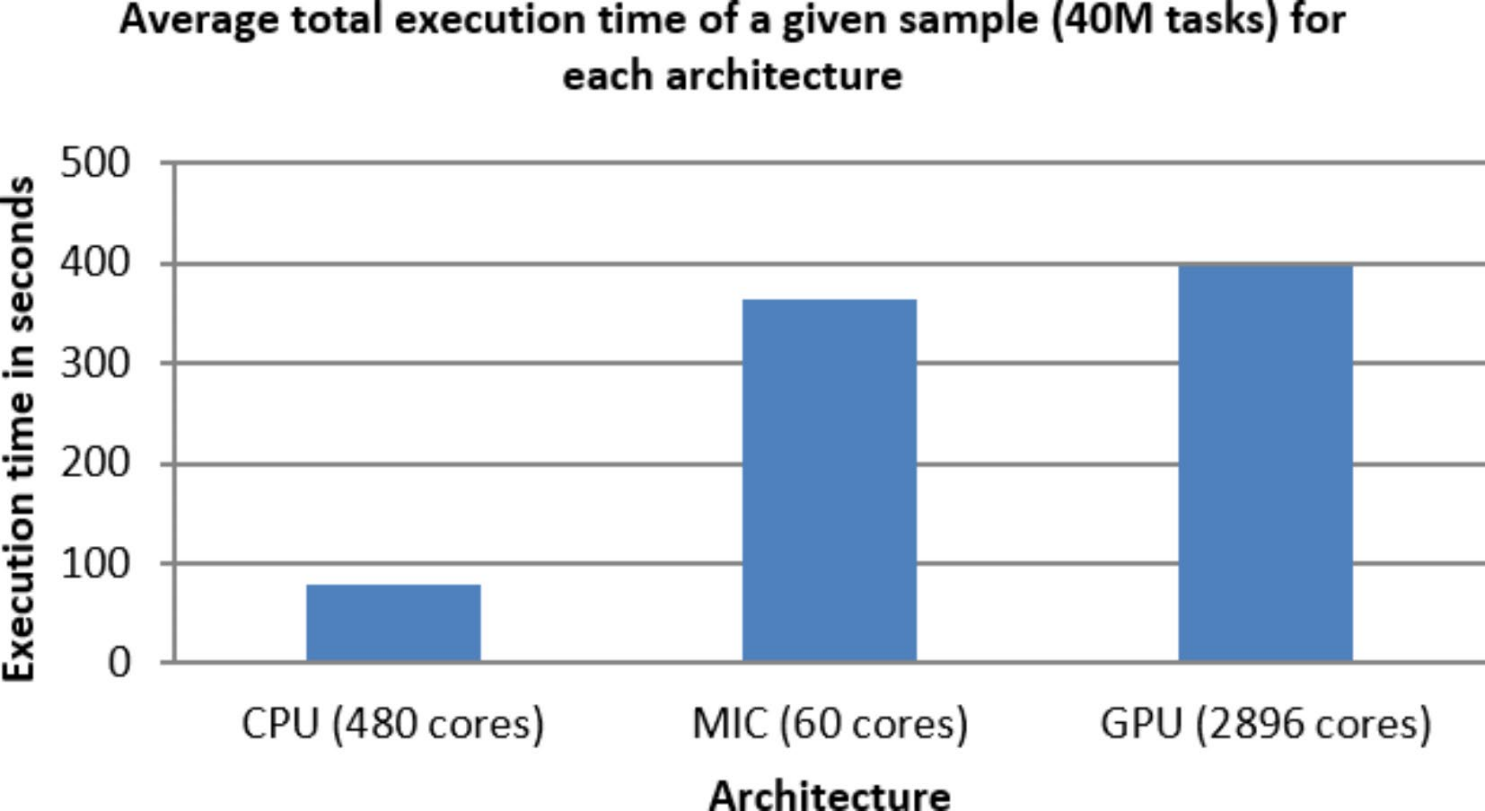
Average total execution time of a given sample (40 M tasks).

**Fig. 12:**
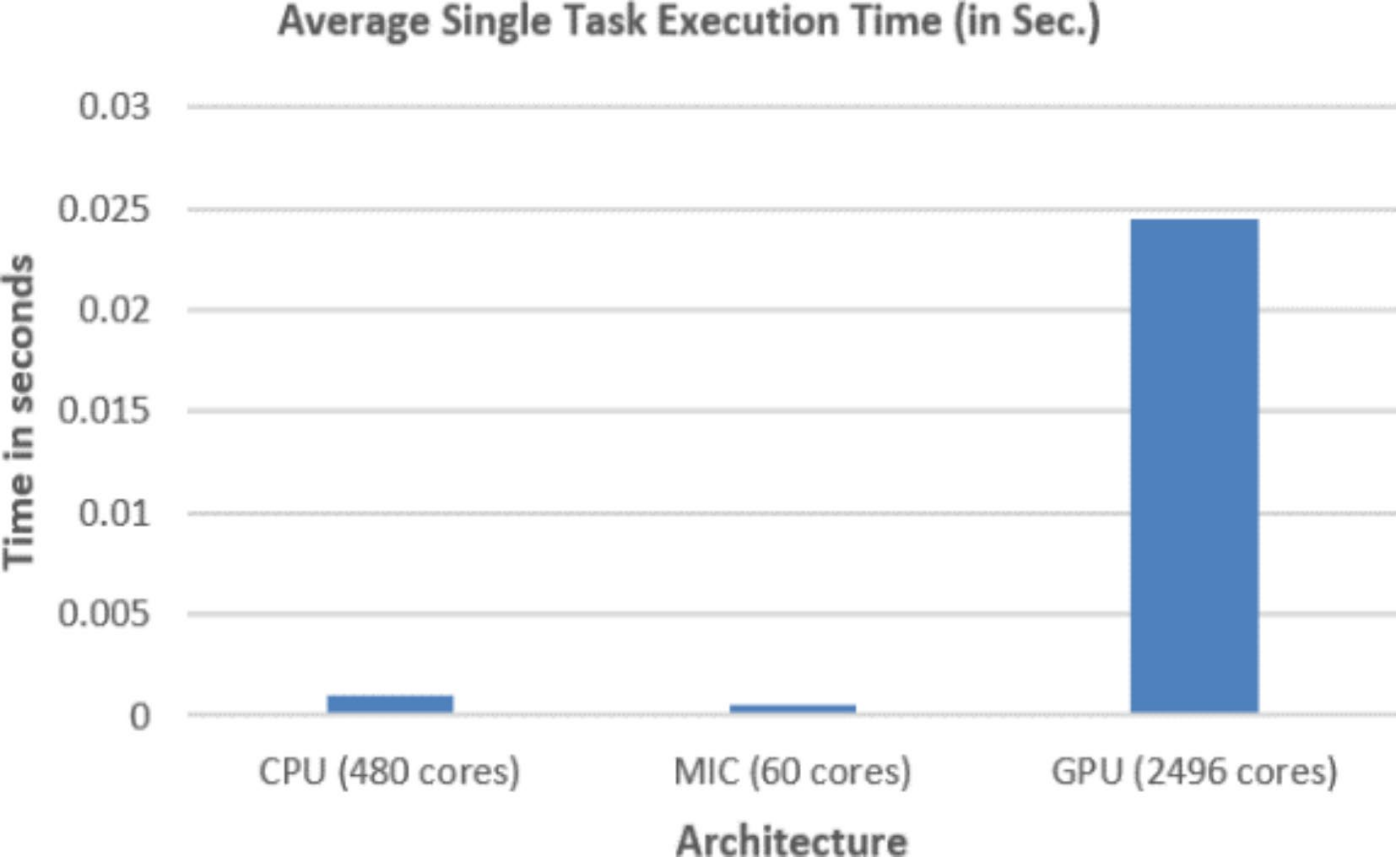
Average single task execution time per core of a given sample (40 M tasks).

Table 7 represents both average total execution time of all (40 M) tasks and actual execution time for a single task for CPU (480 cores), MIC (60 cores) and GPU (2496 cores) architectures. These data are displayed in Figs 11,12 respectively. While MIC architecture has the smallest single task execution time, CPU architecture has the smallest total execution time for the same sample size. This is because of the hardware availability of 480 CPU cores that lead to finish 40 M tasks in less time, however the MIC architectures is faster but we are limited by the number of MIC Cards, on Fig. 12 we are showing single task execution time that shows MIC is faster but we do not have more MIC cards. Accordingly, instead of using execution time of a single task, we consider total execution time of a given predefined sample (40 M) tasks to sort architectures in ascending order.

By employing these data collection and measurement, we aim to obtain accurate and consistent experimental data for each architecture and problem size. These data serve as the foundation for the subsequent steps of our research, including the development and evaluation of the architecture-aware scheduling approach.

## Results validation

In this section, we describe how the experimental results are inputted into the architecture-aware scheduling algorithm to optimize resource utilization. We apply results in "Experimental Results" as inputs to the proposed algorithm to validate the scheduling algorithm based on the experimental results.

**Fig. 13:**
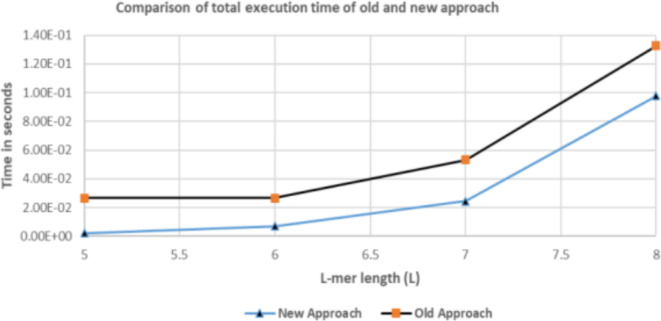
Comparison of total execution time of scheduling algorithm [8], [9] and new approach for L= 5,6,7, and 8.

**Fig. 14:**
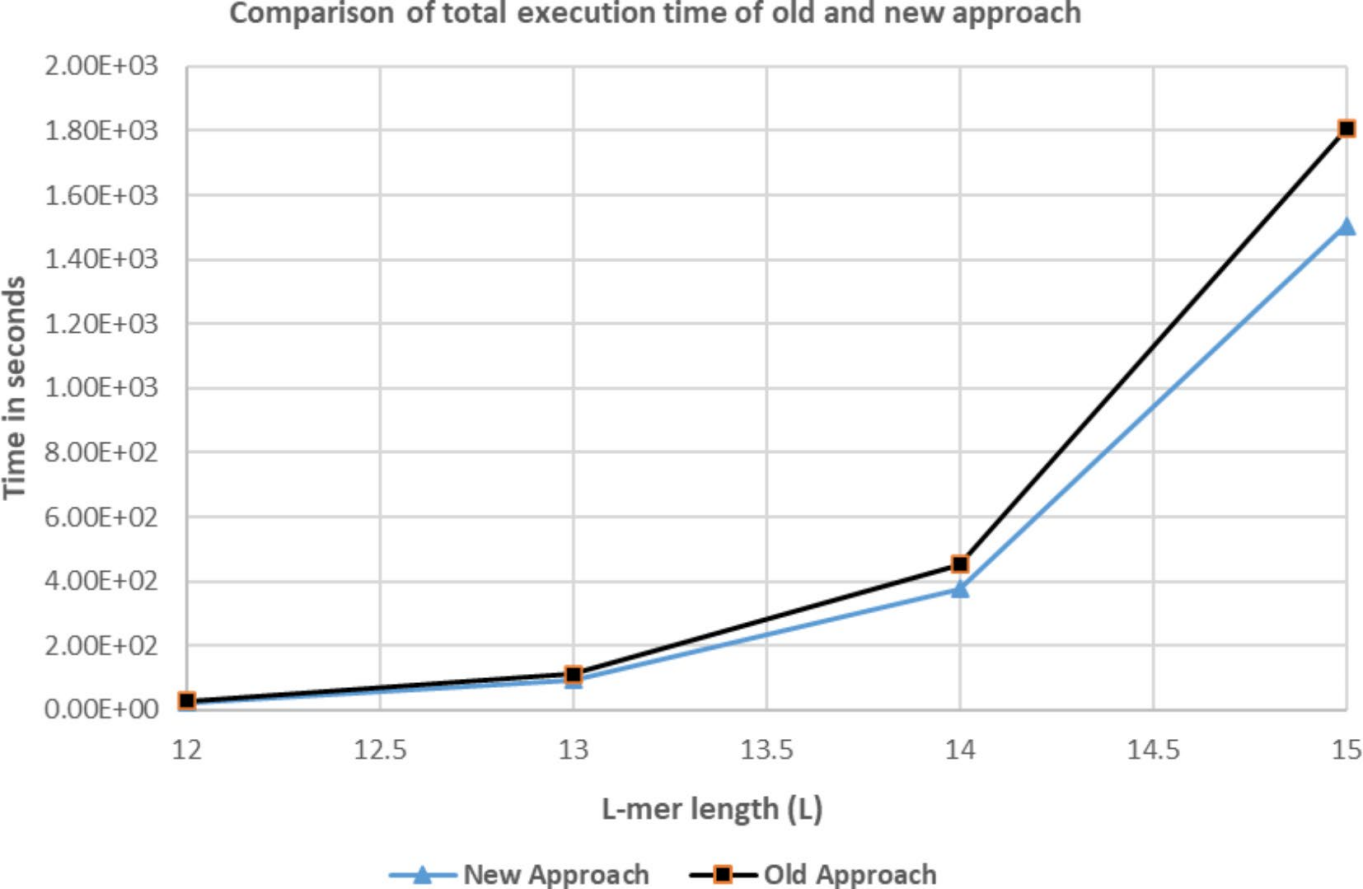
Comparison of total execution time of scheduling algorithm^8,9^ and new approach for L= 12,13,14, and 15.

For a given length $\:\left(\:L\:\right)\:$of L-mer, we get the total number of tasks $\:C=\:{4}^{L\:}\:$in case of Brute Force. These tasks are divided between eligible architectures according to their weight. Table 8 represents ratios of assigned tasks to these eligible architectures according to the proposed scheduling algorithm. Results according to scheduling algorithm^8,9^ are shown in Table 9. In algorithm^8,9^, execution time of first 1 M tasks taken as a reference for all architectures. In addition, algorithm^8,9^ doesn’t consider the new execution time ($\:{T}^{{\prime\:}}$) of hybrid architecture to exclude illegible architectures. Figures 13,14 display a comparison of total execution time between scheduling algorithm^8,9^ and the proposed approach. In Fig. 13 we examine L-mer of lengths 5,6,7 and 8. In Fig. 14 we examine L-mer of length 12,13,14, and 15.

**Table 8:** Represents ratios of assigned tasks applying the proposed scheduling algorithm.

L	# of tasks (c = 4^L)	Architecture name			Total execution time in seconds
		CPU/xeon (480) cores	MIC/xeon phi(60) cores	GPU/K20 (2496) Cuda cores	
5	1024	82%	18%	0%	2.1768E-03
6	4096	82%	18%	0%	7.0746E-03
7	16,384	70.41%	15.45%	14.14%	2.4475E-02
8	65,536	70.41%	15.45%	14.14%	9.7901E-02
9	262,144	70.41%	15.45%	14.14%	3.9160E-01
10	1,048,580	70.41%	15.45%	14.14%	1.4930E + 00
11	4,194,304	70.41%	15.45%	14.14%	5.8985E + 00
12	16,777,216	70.41%	15.45%	14.14%	2.3521E + 01
13	67,108,864	70.41%	15.45%	14.14%	9.4059E + 01
14	268,435,456	70.41%	15.45%	14.14%	3.7616E + 02
15	1,073,741,824	70.41%	15.45%	14.14%	1.5046E + 03

**Table 9:** Represents ratios of assigned tasks applying the scheduling algorithm^8,9^.

L	# of tasks (c = 4^L)	Architecture name			Total execution time in seconds
		CPU/xeon (480) cores	MIC/xeon phi(60) cores	GPU/K20 (2496)Cuda cores	
5	1024	67.2782%	17.0757%	15.6461%	2.6542E-02
6	4096	67.2782%	17.0757%	15.6461%	2.6542E-02
7	16,384	67.2782%	17.0757%	15.6461%	5.3084E-02
8	65,536	67.2782%	17.0757%	15.6461%	1.3271E-01
9	262,144	67.2782%	17.0757%	15.6461%	4.5121E-01
10	1,048,580	67.2782%	17.0757%	15.6461%	1.7783E + 00
11	4,194,304	67.2782%	17.0757%	15.6461%	7.0601E + 00
12	16,777,216	67.2782%	17.0757%	15.6461%	2.8240E + 01
13	67,108,864	67.2782%	17.0757%	15.6461%	1.1288E + 02
14	268,435,456	67.2782%	17.0757%	15.6461%	4.5150E + 02
15	1,073,741,824	67.2782%	17.0757%	15.6461%	1.8060E + 03

Investigating results in Tables 8,9 and Figs 13,14, considering results of total execution time, the proposed algorithm enhances overall system performance and job completion times. For L = 5 and L = 6, All tasks are executed only by both CPU and MIC architectures keeping minimized job completion times. In addition, GPU architecture is idle and can be used in another useful calculations In case of small number of tasks as in row 1 and row 2 in Table 8, the GPU stream multiple processors doesn’t have sufficient workload to hide the memory latency due to the limited bandwidth of GPU memory. The scheduler decided to ignore the GPU availability as the MIC and CPU group finish all the tasks before the GPU finish a single task. This allows other users to make use of the GPU with other problem (application) that best fit GPU architecture.

**Fig. 15:**
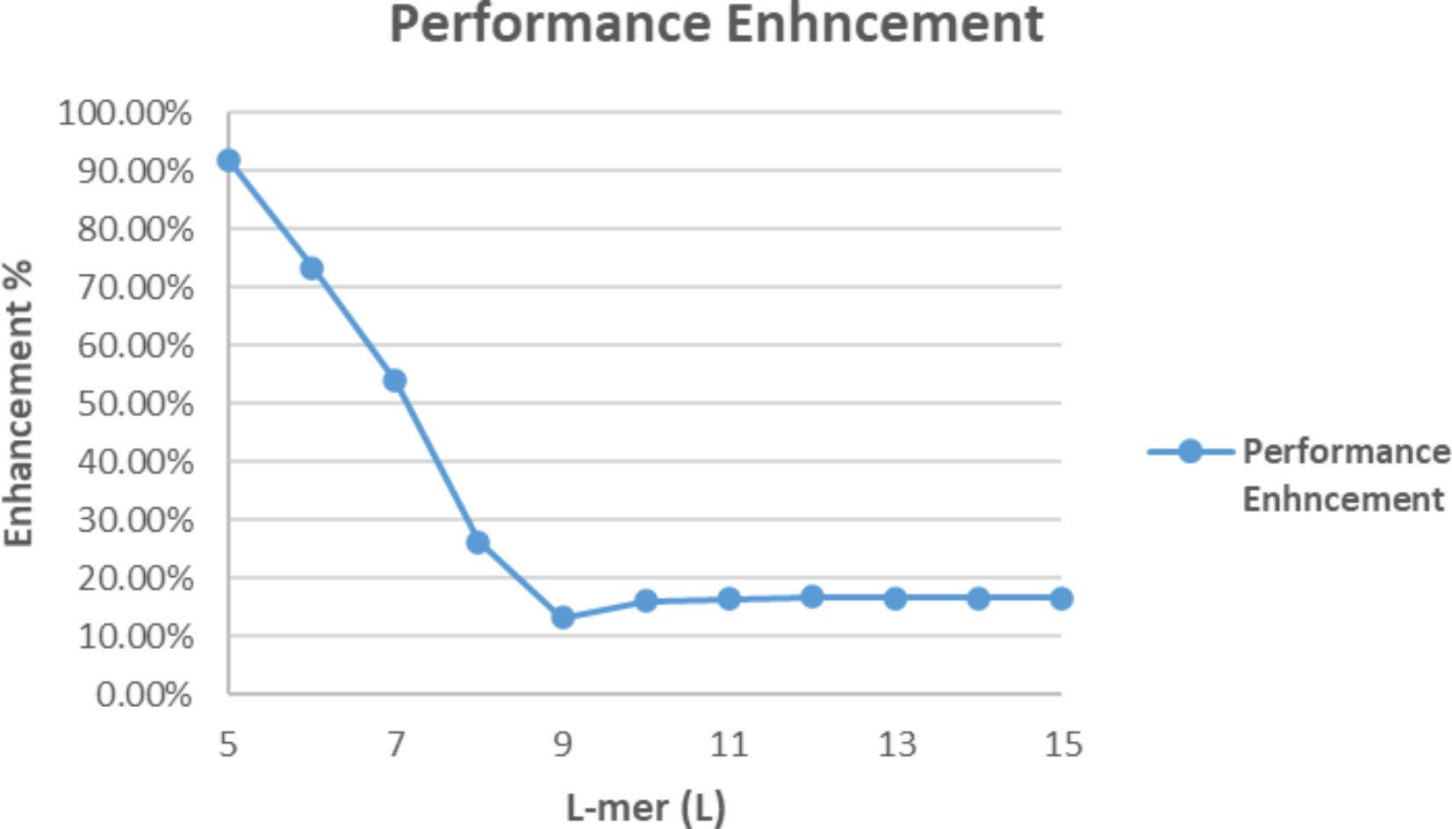
Performance Enhancement.

Table 10 represents percentage of performance enhancement which is displayed in Fig. 15.

**Table 10:** Percentage of performance enhancement.

L	# of tasks (c = 4^L)	Total execution time in seconds		Enhancement %
		Scheduling Algorithm^8,9^	New approach	
5	1024	2.18E-03	2.65E-02	91.80%
6	4096	7.07E-03	2.65E-02	73.35%
7	16,384	2.45E-02	5.31E-02	53.89%
8	65,536	9.79E-02	1.33E-01	26.23%
9	262,144	3.92E-01	4.51E-01	13.21%
10	1,048,580	1.49E + 00	1.78E + 00	16.04%
11	4,194,304	5.90E + 00	7.06E + 00	16.45%
12	16,777,216	2.35E + 01	2.82E + 01	16.71%
13	67,108,864	9.41E + 01	1.13E + 02	16.67%
14	268,435,456	3.76E + 02	4.52E + 02	16.69%
15	1,073,741,824	1.50E + 03	1.81E + 03	16.69%

Considering results in Table 10 and Fig. 15, the proposed approach makes performance enhancement with 16.7% for large number of tasks where $\:(L\ge\:9$), while performance enhancement is increased as length of L-mer decrease to be about 92% for $\:(L=5)$.

We assume that we use all available 20 CPU nodes. In case of downsizing the availability of CPU nodes, Table 11 represents average total execution time of (40 M) tasks and average execution time of a single task for different size of CPU, MIC and GPU architecture. Results shows that single GPU will always outperform single compute node that has 24 CPU cores, however due to the availability of 20 CPU nodes and a single GPU node in our cluster, the scheduler decided to group those 20 CPU nodes into a single compute resource. However, if the available number of CPU nodes drops to 3 nodes, GPU will outperform CPU architecture and the scheduler will consider MIC as first resource, GPU as second resource and CPU as third resource during the scheduler iteration. Where we sort different architectures $\:{A}_{1},{A}_{2},\dots\:,{A}_{p}\:$according to total execution time for a predefined number of tasks $\:{\varvec{C}}^{{\prime\:}}$ (respectively in ascending order) where $\:{T}_{1}<{T}_{2}<\dots\:<{T}_{p}$ as displayed in Fig. 2.

In proposed scheduling algorithm we overcome the following drawbacks of scheduling algorithm^8,9^:

1-

1. Execution time of a single task in CPU (single node) is taken as a reference for CPU architecture. Where results assure that actual execution time of CPU (20 nodes) should be considered.
2. Scheduling algorithm^8,9^ considers execution time of sample with size of 1 M tasks to get single task execution time, while results shows that we should consider sample of size 40 M tasks to get single task execution time with the lowest variance possible in all architectures.
3. The execution time of a single task is calculated by dividing total execution time by total number of tasks of a given sample. This ignores the effect of parallel processing. For the given architecture, the actual execution time of a single task should be calculated by dividing total execution time of a given sample by the number of assigned tasks per each core.
4. In scheduling algorithm^8,9^, Total execution time ($\:T$) of the fastest architecture ($\:{A}_{1}$) is taken as static reference for comparison process to investigate eligible architectures.

Considering that each time we add eligible architecture, we get a new total execution time ($\:{T}^{{\prime\:}}$) of hybrid architecture where ($\:{T}^{{\prime\:}}<T)\:$. Investigating eligible architecture should be done by comparing execution time of a single task of that architecture with the new total execution time $\:{(T}^{{\prime\:}})$ of hybrid architecture. We consider $\:\left({T}^{{\prime\:}}\right)$ in the proposed approach. The proposed approach results in reduced job completion times and improved overall system responsiveness. It enhances the performance with 16.7% for large data size.

**Table 11:** Average total (40 M) and single task execution time (sec) for different size of CPU, MIC and GPU architectures.

#Tasks	Architecture								
	CPU / xeon							MIC / xeon phi (60) cores	GPU /K20 (2496) Cuda cores
	1 node (24) cores	2 nodes (48) cores	3 nodes (72) cores	4 nodes (96) cores	5 nodes (120) cores	10 nodes (240) cores	20 nodes (480) cores		
4.E + 07	1439.1	732	491	373	298.7	152.2	79.6	362.8	396.5
1	8.63E-04	8.78E-04	8.84E-04	8.95E-04	8.96E-04	9.13E-04	9.550E-04	5.442E-04	2.447E-02

## Conclusion

In this study, we proposed an architecture-aware scheduling approach to optimize resource utilization and minimize job completion times for large-scale problems in high-performance computing environments. The research aimed to address the challenges associated with heterogeneous architectures, leading to inefficient resource utilization and extended job completion times. To achieve our objectives, we first conducted a series of experiments using various architectures and different problem sizes to deduce the optimum sample size to calculate average execution time of single task. Based on these findings, we developed the proposed scheduling strategy that maps computational tasks to architectures based on their actual execution time of single task and new total execution time $\:{(T}^{{\prime\:}})\:$ of hybrid eligible architectures.

Our evaluation of the proposed approach demonstrated several key findings. First, the architecture-aware scheduling approach significantly improved resource utilization compared to scheduling algorithm^8,9^. By assigning tasks to architectures with better performance for each specific problem size, the approach achieved higher CPU and GPU utilization. Second, the proposed approach consistently minimized job completion times across various problem sizes and MFP as a benchmark application. The proposed task scheduling led to faster job completion and improved system efficiency. While scheduling algorithm^8,9^ uses total execution time $\:\left(T\right)$ of fastest architecture as a static reference for comparison process to eliminate ineligible architecture, we assure that the new resulting actual total execution time $\:{(T}^{{\prime\:}})\:$ of the hybrid eligible architecture should be used in the comparison process instead. In addition, the actual execution time for a single task should be considered for each architecture. The approach consistently outperformed traditional scheduling methods, demonstrating its relevance and effectiveness in real-world high-performance computing scenarios. We examine the performance trends of each architecture across different problem sizes. This analysis helps us understand how each architecture scales with increasing problem complexities. Identifying the performance trends allows us to determine the architecture’s efficiency in handling a wide range of computational workloads. We investigate the impact of task size on execution times for each architecture. By analyzing how execution times vary with different task sizes, we gain insights into the architecture’s strengths and weaknesses in handling small, medium, and large tasks. Future research directions are proposed to improve the architecture-aware scheduling approach further. These directions include exploring energy-aware scheduling into the scheduling approach. Energy-efficient scheduling aims to minimize energy consumption while maintaining high-performance levels. By incorporating energy-awareness, the approach can contribute to more sustainable and environmentally friendly computing practices.

## Data Availability

Data availabilityData is provided within the manuscript.
